# Supplementary material for: Efficiency fluctuations and noise induced refrigerator-to-heater transition in information engines
Source: Nat Commun. 2020 Feb 21;11:1012. doi: 10.1038/s41467-020-14823-x (PMC7035421; doi:10.1038/s41467-020-14823-x)
Supplement: Supplementary file 1 — Supplementary Information [file 41467_2020_14823_MOESM1_ESM.pdf]

Supplementary Information

Efficiency fluctuations and noise induced refrigerator-to-heater transition in information engines

Paneru et al.

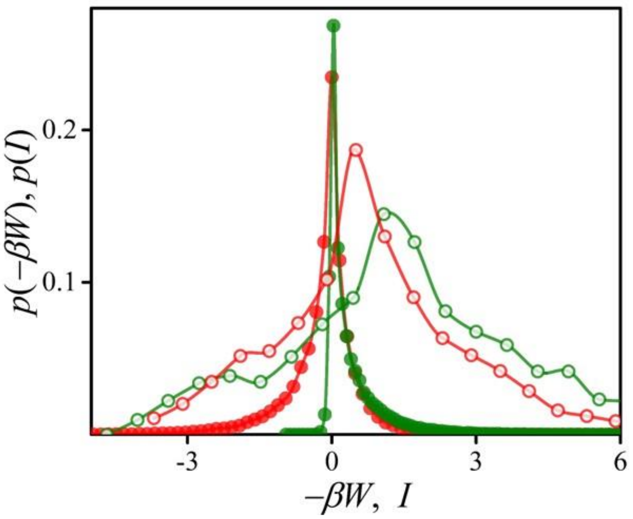

Supplementary Figure 1. Measurement of fluctuations in mutual information and work. Plot of probability distribution functions of mutual information I (open circles) and extracted work $-\beta W$ (filled circles) for cycle period $\tau = 3$ ms and error-level $N/S = 0.06$ (olive) and 0.53 (red). The solid curves are guides to the eyes.

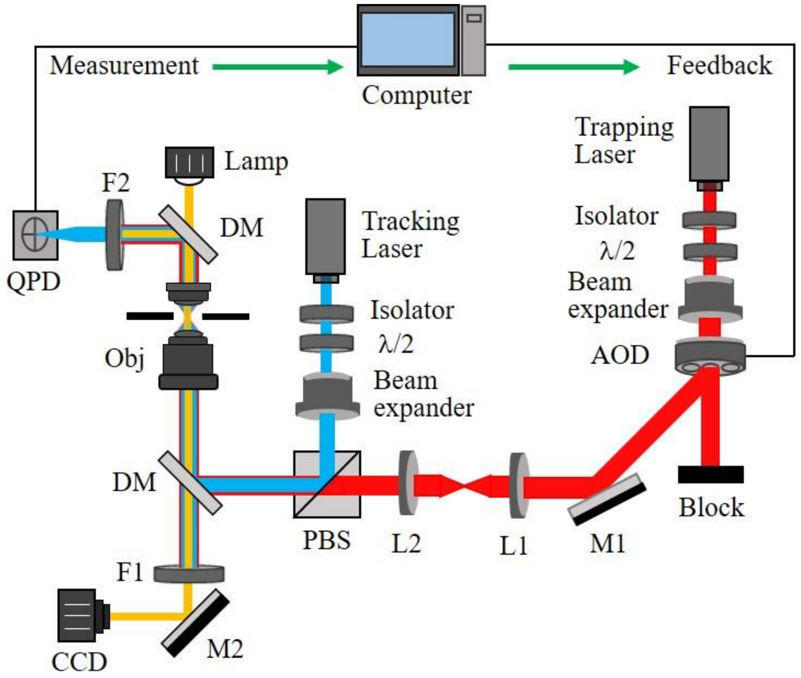

Supplementary Figure 2. Schematics of the optical tweezers set up for realizing colloidal information engine[1]. $\lambda/2$: half Waveplate; L1, L2: lens; DM: dichroic mirror; M1, M2: mirror; PBS: polarizing beam splitter; F1, F2: filter.

Supplementary Note 1: Theoretical description of the model

In this appendix, we show the detail derivation of the analytical model that describes our experiment. We consider a one-dimensional motion of a colloidal particle in a harmonic trap $V(x,t) = \frac{1}{2}k(x-\lambda(t))^2$, where x is the position of the particle, k is a trap stiffness, and $\lambda(t)$ denotes a time-dependent potential center with $\lambda(0) = 0$. The particle is subject to following periodic measurement-feedback-relaxation operations (Fig. 1). In each cycle: 1) the particle position is measured (which is an instantaneous process), 2) the trap center is instantaneously shifted to that position and 3) the particle is then allowed to relax for time τ. The dynamics of the particle during relaxation is described by the overdamped Langevin equation

$$\gamma \frac{dx}{dt} = -k(x-\lambda(t)) + \xi(t), \tag{1}$$

where γ is the dissipation coefficient and ξ is the thermal noise due to the heat bath with temperature T satisfying $\langle \xi(t) \rangle = 0$ and $\langle \xi(t)\xi(t') \rangle = 2\gamma k_{\mathrm{B}} T \delta(t-t')$.

The Fokker Planck equation corresponding to the Supplementary Equation (1) has a Seifert's Gaussian ansatz[2]

$$p(x) = G(x,b(t),S(t)) = \frac{1}{\sqrt{2\pi S(t)}} \exp\left(-(x-b(t))^2 / 2S(t)\right), \tag{2}$$

where $b(t)$ is the mean and $S(t)$ is the variance of the particle position distribution. The dynamics of $b(t)$ and $S(t)$ are obtained by plugging Supplementary Equation (2) into Fokker Planck equation corresponding to the Langevin equation in Supplementary Equation (1)

$$\dot{b}(t) = [\lambda(t) - b(t)], \tag{3}$$

$$\dot{S}(t) = 2[1 - S(t)]. \tag{4}$$

These have solutions

$$b(t) = b(0)\exp(-t/\tau_{\mathrm{R}}) + \lambda(t)(1-\exp(-t/\tau_{\mathrm{R}})), \tag{5}$$

$$S(t) = S + (S(0) - S)\exp(-2t/\tau_{\mathrm{R}}). \tag{6}$$

where $S = k_{\mathrm{B}}T/k$ is the variance of the equilibrium distribution.

We now implement above equations for cyclic information engine. The ith engine cycle of this engine operates by measuring the particle position x_i with respect to the potential center λ_{i-1} to obtain the outcome y_i. Here, the measurement involves a Gaussian noise $p(y|x) = G(x,y,N)$ of variance N. The potential center is then shifted instantaneously to y_i. We next wait for time τ during which the particle relaxes in the shifted potential center λ_i

and the same feedback protocol is repeated for another cycle. We use the subscript '–' for the quantities before measurement and '+' after measurement. The probability distribution function (PDF) of the particle position just before the measurement is given from Supplementary Equation (2) as

$$p_i(x) = G(x, b_i^-(t), S_i^-(t)). \tag{7}$$

The distribution of the measurement outcome y is given by [2,3]

$$p_i(y) = G(y, b_i^-(t), N + S_i^-(t)). \tag{8}$$

Just after measurement, the distribution of x follows

$$p_i(x|y) = G(x, b_i^+(t), S_i^+(t)), \tag{9}$$

with

$$b_i^+(t) = (y_i S_i^-(t) + b_i^-(t)N) / (S_i^-(t) + N), \tag{10}$$

and

$$S_i^+(t) = S_i^-(t)N / (S_i^-(t) + N). \tag{11}$$

Supplementary Note 2: Coordinate transformation

We now perform above calculations in relative frame, where in contrast to the original dynamics, the trap center is fixed at the origin. Instead, the particle is instantaneously shifted by the amount $-y$ during the feedback. Thus, in relative frame of reference, the variance of the particle position remains unchanged and the mean in Supplementary Equation (5) changes to

$$b_i(t) = b_i(0)\exp(-t/\tau_{\mathrm{R}}), \tag{12}$$

Similarly, Supplementary Equation (10) become

$$b_i^+(t) = b_i^-(t)N / (S_i^-(t) + N), \tag{13}$$

The probability distribution of the particle position just after resetting is same as the error distribution,

$$p_{\mathrm{reset}} = G(x, 0, N). \tag{14}$$

Steady state- For cyclic process where a large number of feedback cycles are allowed, the system is assumed to be in steady state. The PDF of the particle position after the relaxation in $i-1$ cycle is same as the PDF before measurement at the start of cycle i. The particle is always reset at the origin at the beginning of relaxation, thus $b_i(0) = 0$ from Supplementary Equation (14). At the end of relaxation, we get $b_i(\tau) = b_i(0)\exp(-\tau) = 0$ from Supplementary Equation (12). Thus in steady state, we get trivially

$$b_i(\tau) = b_i(0) = b^* = 0. \tag{15}$$

Here, * refers to steady state. Similarly, the variance of the PDF in steady state at the start of relaxation is obtained from Supplementary Equation (14) as $S^*(0) = S_i(0) = N$. Using Supplementary Equation (13), the steady state variance at the end of relaxation (or just before measurement) is given by[2,3]

$$S^*(\tau) = S + (N - S)\exp(-2\tau/\tau_{\mathrm{R}}). \tag{16}$$

The interesting limiting cases are-

(i) Error-free measurements, $N \to 0,$ in which case $S^* \to S(1 - \exp(-2\tau/\tau_{\mathrm{R}}))$.

(ii) Equilibrium, $\tau \to \infty,$ for which $S^* \to S.$

(iii) Additionally, when $N = S,$ we obtain an interesting noise-driven-equilibrium limit, $S^*(t) = S$ for all $\tau.$

Finally, the steady state PDFs of the particle position before and after the measurements can be obtained by using Supplementary Equations (15) and (16) in Supplementary Equations (7-9)

$$p(x) = G(x, 0, S^*) \tag{17}$$

$$p(x|y) = G\left(x, \frac{yS^*}{S^* + N}, \frac{NS^*}{S^* + N}\right) \tag{18}$$

$$p(y) = G(y, 0, S^* + N) \tag{19}$$

Supplementary Note 3: Thermodynamics of the engine

The average work performed on the system per cycle in steady state is given by

$$\langle \beta W \rangle = \frac{\beta k}{2} \int dxdyp(x|y)p(y)[(x-y)^2 - x^2] = \frac{1}{2} \cdot \frac{N - S^*}{S}. \tag{20}$$

with standard deviation of $\mathrm{std}(\beta W) = \sqrt{1/2(N^2 + (S^*)^2)/S^2}$. The average heat supplied to the system during the relaxation is given as sum of system entropy change $-\int dxp(x)\ln p(x) = \ln(S*(\tau)/S(0))$ and total entropy change $\int_0^\tau (\dot{b}^2 + \dot{S}^2)dt = \ln(S^*(\tau)/S(0)) - 1/2\ln[(S^*(\tau))^2 - S^2(0)],$

$$\langle \beta Q \rangle = -\frac{1}{2} . \frac{N - S^*}{S}. \tag{21}$$

Similarly, the steady state average mutual information gain immediately after the measurement is given by

$$\langle I \rangle = \int dxdyp(x|y)p(y)\ln[p(x|y)/p(x)] = \frac{1}{2}\ln\left(1+\frac{S^*}{N}\right). \tag{22}$$

Also, the fluctuation in mutual information is given by $\mathrm{std}(I) = \sqrt{S^*/(S^*+N)}.$ Using the steady state probabilities in Supplementary Equations (17-19) and Ref.[4,5], we obtain following expression for the generalized integral fluctuation theorem

$$\begin{aligned}\langle \exp(-\beta(W-\Delta F)-\Delta I)\rangle &= \int dxdyp(x)p(y)\exp\left(-\left((x-y)^2-x^2\right)/2\right) \\ &= \left[1+\left(1-\frac{S^*}{S}\right)\left(\frac{N+S^*}{S}\right)\right]^{-1/2},\end{aligned} \tag{23}$$

which is equal to unity when $S^* = S,$ i.e. when the system is fully relaxed at the end of each cycle.

Supplementary References

1 Paneru, G. *et al.* Optimal tuning of a Brownian information engine operating in a nonequilibrium steady state. *Physical Review E* **98**, 052119, doi:10.1103/PhysRevE.98.052119 (2018).

2 Abreu, D. & Seifert, U. Extracting work from a single heat bath through feedback. *EPL (Europhysics Letters)* **94**, 10001 (2011).

3 Bauer, M., Abreu, D. & Seifert, U. Efficiency of a Brownian information machine. *Journal of Physics A: Mathematical and Theoretical* **45**, 162001 (2012).

4 Sagawa, T. & Ueda, M. Generalized Jarzynski Equality under Nonequilibrium Feedback Control. *Physical Review Letters* **104**, 090602 (2010).

5 Parrondo, J. M. R., Horowitz, J. M. & Sagawa, T. Thermodynamics of information. *Nature Physics* **11**, 131, doi:10.1038/nphys3230 (2015).
